# Effect of Neonatal Asphyxia on the Impairment of the Auditory Pathway by Recording Auditory Brainstem Responses in Newborn Piglets: A New Experimentation Model to Study the Perinatal Hypoxic-Ischemic Damage on the Auditory System

**DOI:** 10.1371/journal.pone.0126885

**Published:** 2015-05-26

**Authors:** Francisco Jose Alvarez, Miren Revuelta, Francisco Santaolalla, Antonia Alvarez, Hector Lafuente, Olatz Arteaga, Daniel Alonso-Alconada, Ana Sanchez-del-Rey, Enrique Hilario, Agustin Martinez-Ibargüen

**Affiliations:** 1 Research Unit on Experimental Perinatal Physiopathology, Cruces University Hospital, Barakaldo, 48080, Bizkaia, Spain; 2 Department of Cell Biology and Histology, Faculty of Medicine and Dentistry, University of the Basque Country, Barrio Sarriena s/n, Leioa, 48940, Bizkaia, Spain; 3 Department of Otorhinolaryngology, Basurto University Hospital, Faculty of Medicine, University of the Basque Country, Barrio Sarriena s/n, Leioa, 48940, Bizkaia, Spain; University of Naples Federico II, ITALY

## Abstract

**Introduction:**

Hypoxia–ischemia (HI) is a major perinatal problem that results in severe damage to the brain impairing the normal development of the auditory system. The purpose of the present study is to study the effect of perinatal asphyxia on the auditory pathway by recording auditory brain responses in a novel animal experimentation model in newborn piglets.

**Method:**

Hypoxia-ischemia was induced to 1.3 day-old piglets by clamping 30 minutes both carotid arteries by vascular occluders and lowering the fraction of inspired oxygen. We compared the Auditory Brain Responses (ABRs) of newborn piglets exposed to acute hypoxia/ischemia (n = 6) and a control group with no such exposure (n = 10). ABRs were recorded for both ears before the start of the experiment (baseline), after 30 minutes of HI injury, and every 30 minutes during 6 h after the HI injury.

**Results:**

Auditory brain responses were altered during the hypoxic-ischemic insult but recovered 30-60 minutes later. Hypoxia/ischemia seemed to induce auditory functional damage by increasing I-V latencies and decreasing wave I, III and V amplitudes, although differences were not significant.

**Conclusion:**

The described experimental model of hypoxia-ischemia in newborn piglets may be useful for studying the effect of perinatal asphyxia on the impairment of the auditory pathway.

## Introduction

Hearing, mostly developed over the first few years of life, is a fundamental sense for communication skills. In this sense, a healthy auditory system from birth is needed to develop correct hearing and avoid the language delays associated with perinatal hearing loss. Otherwise, perinatal hearing loss will be associated with language delay. At the same time, the maduration of the auditory system finishes at 18 months and is related to the process of myelination of the central nervous system. Normal development, maduration and myelination of the auditory system can be impaired by certain pathological conditions such as viral infections, hyperbilirubinemia, meningitis or perinatal asphyxia and is related to the process of myelination of the central nervous system. Specifically, in kernicterus, the auditory system is damaged at the level of the cochlear nuclei and when hypoxia is severe, it is associated with mental retardation and neuromuscular disorders (Lefebvre, et al., 2002; Cao et al., 2010) [1–2].

In this context, the synaptic nuclei of the brainstem are an important part of the auditory system and represent the first synapse of the acoustic nerve. The nerve bundles that innervate the cochlear nuclei are the anatomical substrate that has a decisive role in modulating the acoustic information from afferent and efferent nerves transmitted to the brainstem and higher cortical levels (Rubel and Fritzsch, 2002; Kishan et al., 2011; Appler and Goodrich, 2011) [3–5].

Hypoxia–ischemia (HI) is a major perinatal problem that results in severe damage to the brain. In humans, 80% of the growth of the central nervous system (CNS) takes place in the last two months of gestation and the first few months of life, periods during which this system is particularly damaged by the effects of a low blood flow (du-Plessis and Volpe, 2002; Soehle et al., 2003) [6,7]. In line with this, hypoxic/ischemic brain injury that takes place during this perinatal period is one of the most common causes of long-term severe neurological impairments. (Volpe, 2001; Perlman, 2004) [8,9]. Auditory brainstem evocated potentials are very useful in the diagnosis of these injuries because they can quantify the degree of damage. (Martinez Ibargüen et al., 1993) [10]. Neurons are the elements that are the most vulnerable to oxygen deprivation but if exposure is sufficiently severe other cell systems, in particular the glial cells, are also affected (Hilario et al, 2005; Goñi de Cerio et al, 2007) [11,12]. Further, the immature brain is made more vulnerable by a series of specific factors (Inder and Volpe, 2000, Alvarez A et al. 2007) [13,14], including a greater susceptibility to excitotoxicity and to free radicals and a greater tendency to cell death by apoptosis (Echteler et al., 2005) [15]. So, plasticity of the synaptic connections depends on various factors such as perisynaptic glial cells and pre- and post-synaptic neuronal mechanisms (Todt K.J, et al, 2006) [16].

The newborn piglet is considered an ideal experimental model for studying metabolism and brain circulation. Specifically, the development of the brain of a newborn piglet, < 5 days old, is similar to the development of the brain of a newborn human infant, making it well suited for studying lesions observed in premature infants who have experienced a period of HI. Further, this model allows the use of instruments required to study responses over not just the short (hours) but also the long (days or weeks) term, unlike other animal models given their small size and weight (e.g., the rat fetus). In the newborn piglet model, various methods have been used to induce brain injury, occlusion of the carotid while reducing the inhaled oxygen fraction being the most widely used [17]. In the present work we studied HI piglets up to 360 min post-HI insult. This period is commonly selected in this model because the biochemical, neuropathological, and neurobehavioral consequences of HI are well established over that interval of time [17–19].

The objective of the present study was to develop a new animal experimental model in piglets for studying the effect of perinatal asphyxia on the auditory pathway. For this, we studied the integrity of the brainstem after HI investigating whether such damage produces electrophysiological changes in Auditory Brainstem Response (ABR) recordings.

## Methods

### Animal preparation

The study was performed in the laboratories of the Research Unit on Experimental Perinatal Physiopathology of Cruces Hospital. The experimental protocol met European and Spanish regulations for protection of experimental animals (86/609/EEC and RD 1201/2005) and was approved by the Ethical Committee for Animal Welfare of Cruces Hospital and has been extensively described elsewhere [20].

**Fig 1:**
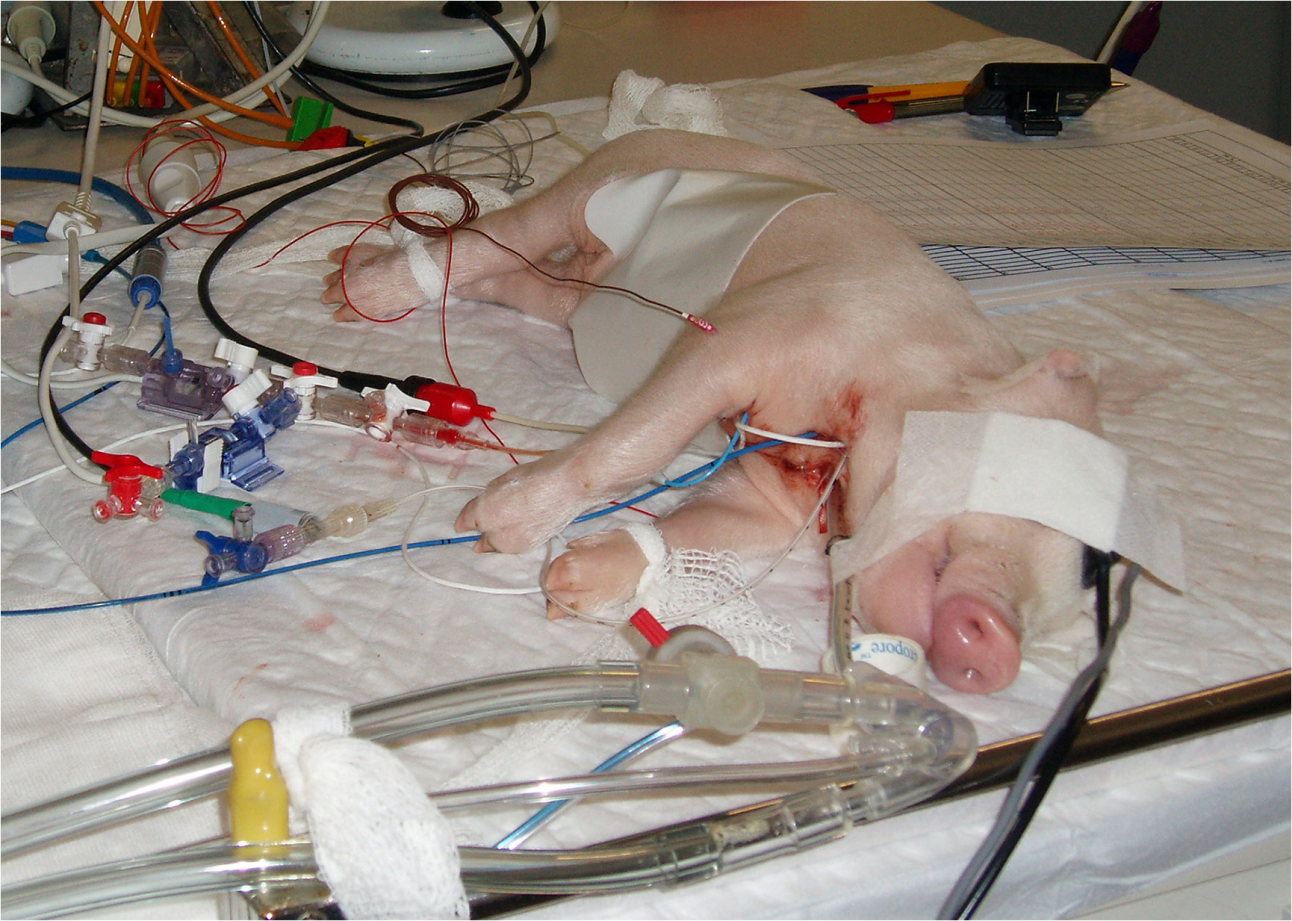
Piglet. Panoramic view of the experimental model in newborn piglet.

Briefly, 1- to 3-d-old piglets, previously anesthetized and paralyzed with a perfusion of fentanyl, propofol and midazolam in dextrose 5% (0.004, 3 and 0.5 mg/kg/h, respectively) and vecuronium (3 mg/kg/h), were intubated and mechanically ventilated (Bourns BP200; CA), Fig 1. The femoral artery was cannulated to monitor blood pressure (Ominare CMS24; HP, Göblingen, Germany) and to obtain blood samples. Blood oxygen saturation was monitored by transcutaneous pulse oximetry. Blood gases and glycemia were regularly checked to adjust the ventilator settings and/or to add dextrose or vasoactive drugs to correct deviations from appropriate levels. Rectal temperature was maintained between 37.5 and 38.5°C with heat lamps.

### Experimental procedure

HI group (n = 6): HI was induced by clamping carotid arteries bilaterally with vascular occluders and lowering the fraction of inspired oxygen to 8–10% over 30 minutes (age 1.3 ± 0.3 d, and weight 1.7 ± 0.1 kg). (Fig 1). Control group (n = 10): Piglets were similarly anesthetized and intubated but were kept with normal oxygen levels and their carotids were not clamped (age 1.7 ± 0.5 d, and weight 1.6 ± 0.1 kg). Animals in each group were kept ventilated with sedation and analgesia up to 6 h after HI. At the end of the experiment, anesthetized piglets were sacrificed with intravenous potassium chloride.

Neurophysiological and neurobehavioral assessment of animals: The tissue oxygenation index expressed as percentage, and normalized tissue Hb index were continuously monitored using a near-infrared spectroscopy (NIRS) system (NIRO-200; Hamamatsu Photonics KK, Joko Cho, Japan). The NIRS sensor was placed on the skull frontoparietally at the midline and fixed with bandages. Brain activity was monitored using a two-channel bedside amplitude-integrated electroencephalography (aEEG) monitor (BRM2; BrainZ Instruments, Auckland, New Zealand) using five needle electrodes placed at C3–P3, C4–P4 and near the shoulder with lead directing towards the head as recommended in the user guide by the manufacturer (International 10–20 system). NIRS and aEEG were continuously recorded throughout the period of mechanical ventilation and sedation and analgesia. The aEEG from each pair of electrodes was processed with a pass-band of 1 Hz to 50 Hz (single pole high-pass filter at 1 Hz, 4-pole low-pass Butterworth filter at 50 Hz) to exclude muscle artifacts. A neurological examination was performed using an adapted standardized approach for scoring piglets [19]. The aEEG from each pair of electrodes was processed with a pass-band of 1 Hz to 50 Hz (single pole high-pass filter at 1 Hz, 4-pole low-pass Butterworth filter at 50 Hz) to exclude artifacts. Table 1 shows aEEG, TOI and nTHI in control and Hypoxic-Ischemic groups.

**Table 1:** Neurophysiological assessment of piglets: amplitude-integrated electroencephalography aEEG, tissue oxygenation index (TOI) and normalized tissue hemoglobin index (nTHI) in control and Hypoxic-Ischemic (HI) groups.

Neurophysiological assessment (mean±SD)	Basal	20 minutes	1 hour	3 hours	6 hours
**aEEG Control (μV)**	21,00 ± 1,10	22,00 ± 1,10	21,00 ± 0,57	22,00 ± 1,23	23,00 ± 1,19
**aEEG HI (μV)**	23,50 ± 1,32	1,92 ± 0,55	2,33 ± 0,57	2,72 ± 0,81	3,12 ± 0,77
**TOI Control (%)**	50,00 ± 0,66	51,67 ± 0,66	52,33 ± 0,95	52,33 ± 1,06	52,67 ± 0,49
**TOI HI (%)**	52,00 ± 1,12	26,63 ± 1,12	50,25 ± 1,42	52,00 ± 0,48	53,29 ± 0.69
**nTHI Control (%)**	95,67 ± 2,94	96,33 ± 3,00	95,00 ± 4,29	93,33 ± 3,53	93,00 ± 5,35
**nTHI HI (%)**	99,13 ± 2,75	122,80 ± 3,93	107,63 ± 2,70	110,00 ± 3,76	104,57 ± 2,24

HI: Hypoxia–ischemia. TOI: tissue oxygenation index. nTHI: normalized tissue hemoglobin index. SD: standard deviation

### Auditory Brainstem Responses (ABR)

A GSI Audera device running software version 1.0.3.4 was used to record auditory steady-state evoked potentials. The measurements were performed in a sound-proofed room to ensure that background noise did not affect our results. The specifications of the acquisition were: sweep time 10 ms with 150 and 3000 Hz filters for low and high frequencies, respectively.

Stimulation was performed by means of clicks at 1000 Hz carrier frequency and 10 stimuli/second repetition rate. The averages were taken of 2006 responses. The study started with continuous clicks at intensities of 75 dB nHL, this being progressively reduced by 20 dB (to 55, 35 and 15 dB nHL) until identification of the auditory threshold.

Repeated auditory brainstem responses (ABRs) were recorded for both ears of all of the animals in the following conditions: 1) baseline, before the start of the experiment; 2) during the stabilization phase of the animal, after the carotid and femoral surgery; 3) After 30 minutes of HI injury, and 4) every 30 minutes during 6 h after the HI injury. Wave components of the responses were analysed to assess brainstem auditory electrophysiology. The parameters analysed were hearing thresholds, peak amplitudes (μV), peak latencies (ms) and interpeak latencies.

### Statistical analysis

SPSS 15.0.0 software was used for all statistical analyses. Data are presented as means ± standard deviation (SD). Median values have been compared using Mann-Whitney U test. A p value < 0.05 was considered to be significant.

### Ethical considerations and limitations

The experimental protocol was overseen by the Ethics Committee for Animal Welfare and the Research Commission of Cruces Hospital in accordance with EU directive 86/609/EEC and the NIH Guide. Once experiments had been completed, animals were sacrificed according to the spanish and european guidelines.

## Results

**Fig 2:**
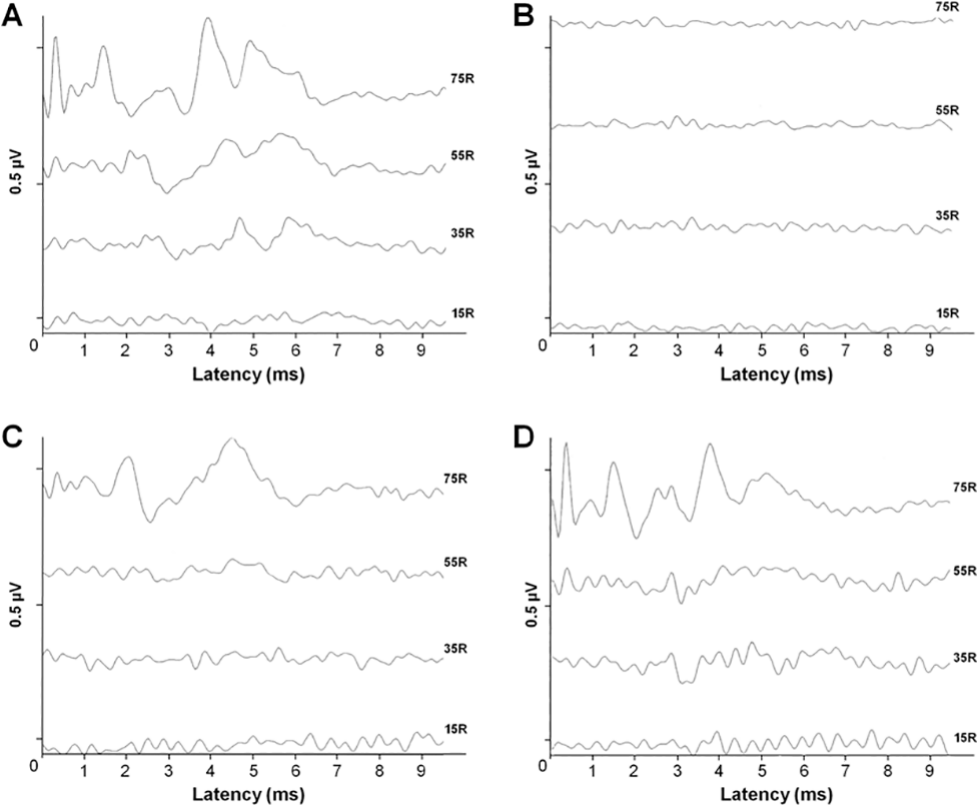
ABRs responses. Acoustic brainstem responses before, during and after hypoxia/ischemia exposure. A) Basaline. B) During HI event. C) 30 min. after HI event. D) 360 min. after HI event. We can observe four different traces associated to different intensities of ABR presentations showing that the responses disappear during exposure, partially recover after 30 minutes and completely recover after 360 minutes.

Fig 2 shows ABR recordings before, during and after inducing HI. It can be seen that the ABRs in newborn piglets in the control group followed a normal pattern, all with the same morphology and thresholds of 15–35 dB. Further, the waves of the ABRs completely disappeared during the HI exposure and start recovering over a period of 30–60 minutes following the end of the HI conditions. 360 minutes after the hypoxic ischemic event, the waves practically return to baseline latency and amplitude.

Table 2 shows the mean and SD of the amplitudes, latencies and intervals for waves I, III and V in the control group and HI groups during and after the event. Diminished amplitudes and increased latencies were observed in the HI group relative to the control group in the first minutes after the HI event, although these differences were not significant in any case, p>0.05. ABR recordings remained steady with no further variations through the 6-hour post-HI period. While I and III waves disappeared during the damage and recovered in the first 30 minutes following the end of the HI conditions, the wave V disappeared in the same point but the recovery occurred after 90 minutes following the end of the damage.

**Table 2:** Means and standard deviations (SD) of amplitudes, latencies and intervals of waves I, III and V in the control group and damaged group during and after the hypoxic ischemic event.

ABR VARIABLES	CONTROL mean ± SD	30 min HI mean ± SD	60 min HI mean ± SD	90 min HI mean ± SD	150 min HI mean ± SD	360 min HI mean ± SD
**I Amplitude (μV)**	0,139 ± 0,025	0,106 ± 0,004	0,130 ± 0,026	0,131 ± 0,033	0,142 ± 0,026	0,148 ± 0,014
**III Amplitude (μV)**	0,182 ± 0,042	0,161 ± 0,011	0,161 ± 0,021	0,162 ± 0,045	0,176 ± 0,027	0,158 ± 0,03
**I Latency (ms)**	1,598 ± 0,16	1,716 ± 0,244	1,66 ± 0,112	1,66 ± 0,272	1,74 ± 0,221	1,58 ± 0,035
**III Latency (ms)**	3,913 ± 0,12	4,366 ± 0,303	3,99 ± 0,214	3,95 ± 0,165	4,09 ± 0,167	3,92 ± 0,208
**V Latency (ms)**	5,056 ± 0,174	-	-	5,46 ± 0,127	5,185 ± 0,2	5,1 ± 0,028
**Interval latency I-III (ms)**	2,315 ± 0,12	2,65 ± 0,152	2,33 ± 0,242	2,293 ± 0,26	2,35 ± 0,121	2,34 ± 0,254
**Interval latency III-V (ms)**	1,142 ± 0,12	-	-	1,58 ± 0,056	1,23± 0,23	1,23 ± 0,188
**Interval latency I-V (ms)**	3,457 ± 0,156	-	-	4,03 ± 0,141	3,65 ± 0,23	3,452 ± 0,218

HI: Hypoxia–ischemia. min: minutes. SD: standard deviation.

**Fig 3:**
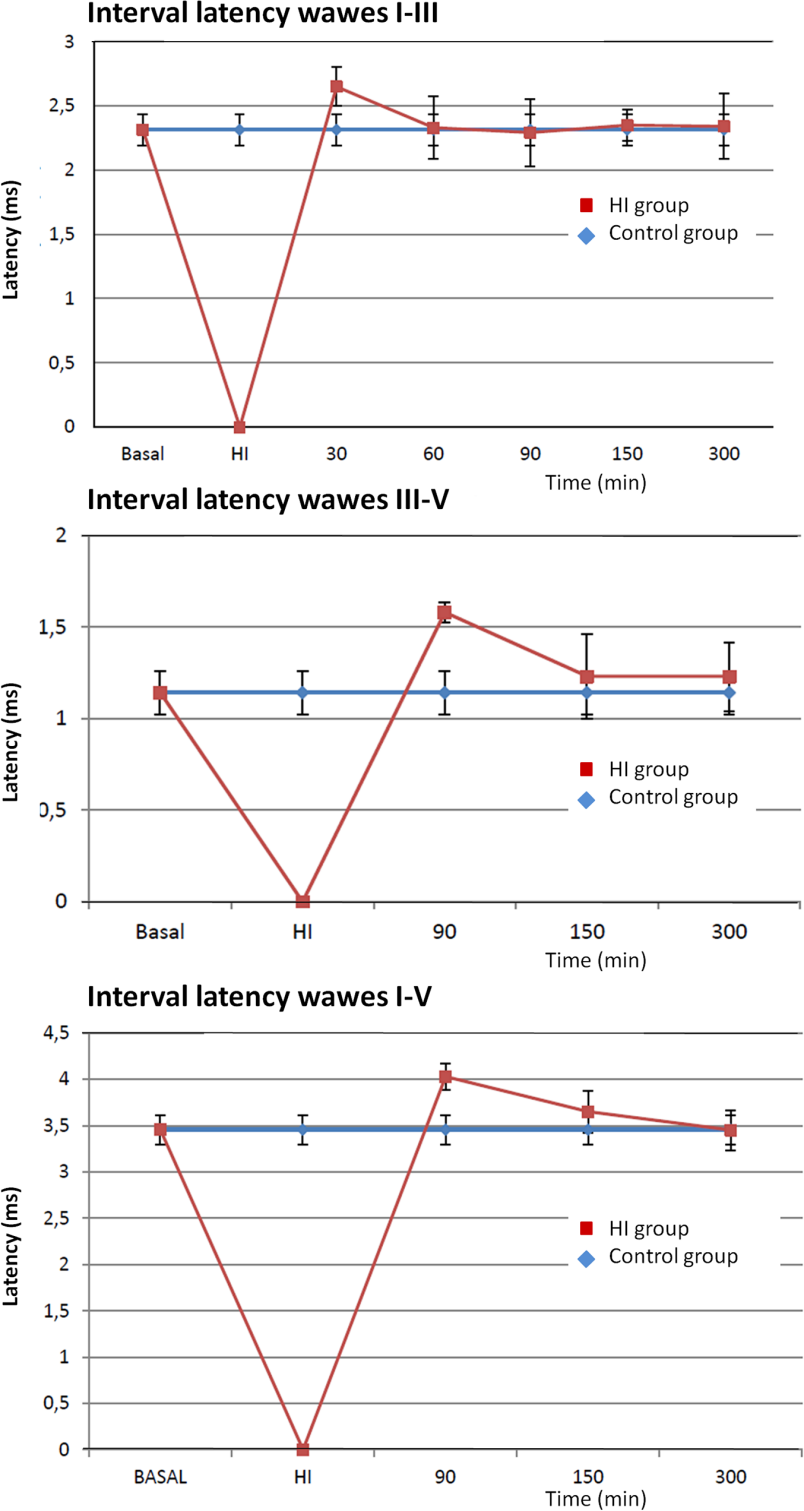
ABRs interval latency. ABRs were recorded for both ears before the start of the experiment (baseline), after 30 minutes of HI injury, and every 30 minutes during 6 h after the HI injury. Time conduction graphics in the interval between the waves I-III (A), III-V (B) and I-V (C) are shown.

Fig 3 shows graphics of the interval latency that represent the time conduction between the wave’s I-III, III-V and I-V in control and HI group. In all the cases, interval latency increased relative to the control group after the HI event, but it returned to baseline in the following hours.

## Discussion

Meyer et al. and Newton authors [21,22] have studied auditory impairment in infants and children, attempting to identify the causes of the impairment. They found that there were often several potential causative factors for the impairment and that it could not be attributed to any one cause. In relation to this, sensory-neural hearing loss (SNHL) is a common feature in the post-asphyxial syndrome in newborns. In this study, we used ABR in newborn piglets to examine brainstem neural conduction. The present work supports the utility of this novel hypoxic-ischemic experimental model to study the effect of perinatal asphyxia on the auditory system.

Previous experiments in animals indicated that a normal ABR requires the integrity of an anatomically diffuse system comprising a set of neurons, their axons, the synapses between them, and the neurons on which each of the ABR components terminate [23, 24]. Disruption of any portion of the system will alter the amplitude and/or the latency of that component.

Jiang et al. [25] compared 68 preterm infants exposed to perinatal HI with healthy preterm infants. The preterm infants after perinatal HI showed a significant increase in the I–V interval, while the III–V interval and III–V/I–III interval ratio also increased significantly. Compared with normal term controls, these preterm infants showed similar, but slightly more significant, abnormalities. These results demonstrated that functional integrity of the brainstem was impaired mainly in the most central part. These findings agree with our results in the newborn piglet model, in which that wave V was more severely affected than waves III and I. These authors concluded that HI damage to the preterm brainstem is unlikely to completely recover in a relatively short period after the insult, which is also in accordance with our findings. In a previous study [26], the same group found I–V and III–V intervals and the III–V/I–III interval ratio increased significantly after perinatal HI compared with values in controls. These findings reflect abnormalities in neural conduction related to neural synchronization, myelination and synaptic function in the HI brainstem [27]. These data provide clear evidence that the functional integrity of the brainstem and auditory system are damaged after perinatal HI and that ABRs detect the impairment. This is in agreement with their previous findings in term infants after HI and animals with experimental HI [27–29].

Smit et al. [30] used an animal model with lambs to study the functional impairment of the auditory pathway after perinatal asphyxia. They observed significantly elevated mean thresholds, diminished amplitudes, and elevated latencies in the asphyxia group relative to the control group through the observation period. They also found that propofol anesthesia reduces auditory impairment after perinatal asphyxia. Their results support the hypothesis that some drugs might prevent the functional changes to the auditory pathway in the event of perinatal asphyxia.

Hence, the model we have developed will facilitate future research into the effect of different treatments for the prevention of injury to the central nervous system. In relation to this, we have recently reported that post-HI administration of cannabinoid cannabidiol to newborn piglets, for example, produces beneficial effects in the very early hours after HI, as reflected by the improvement in brain activity monitored by aEEG and metabolism as measured by NIRS [20]. These beneficial effects are sustained 72 h after HI and they are associated not only with histological improvement but also with neurobehavioral normalization [31–33].

## Conclusions

The hypoxic-ischemic experimental model in newborn piglets is a suited model for studying the functional effects of perinatal asphyxia on the brainstem. Moreover, the development of this model will allow us in the future to develop interventions to minimise the injury during acute episodes study and to study the useful of new strategies for the prevention of cerebral impairment in neonates.
